# Conversion of the BASE Prion Strain into the BSE Strain: The Origin of BSE?

**DOI:** 10.1371/journal.ppat.0030031

**Published:** 2007-03-09

**Authors:** Raffaella Capobianco, Cristina Casalone, Silvia Suardi, Michela Mangieri, Claudia Miccolo, Lucia Limido, Marcella Catania, Giacomina Rossi, Giuseppe Di Fede, Giorgio Giaccone, Maria Grazia Bruzzone, Ludovico Minati, Cristiano Corona, Pierluigi Acutis, Daniela Gelmetti, Guerino Lombardi, Martin H Groschup, Anne Buschmann, Gianluigi Zanusso, Salvatore Monaco, Maria Caramelli, Fabrizio Tagliavini

**Affiliations:** 1 Fondazione I.R.C.C.S. Istituto Neurologico Carlo Besta, Milan, Italy; 2 Istituto Zooprofilattico Sperimentale del Piemonte, Liguria e Valle d'Aosta, Torino, Italy; 3 Istituto Zooprofilattico Sperimentale della Lombardia ed Emilia Romagna, Brescia, Italy; 4 Friedrich-Loeffler-Institut, Institute for Novel and Emerging Infectious Diseases, Greifswald, Insel Riems, Germany; 5 Department of Neurological and Visual Science, Section of Clinical Neurology, Policlinico G. B. Rossi, Verona, Italy; University of Toronto, Canada

## Abstract

Atypical neuropathological and molecular phenotypes of bovine spongiform encephalopathy (BSE) have recently been identified in different countries. One of these phenotypes, named bovine “amyloidotic” spongiform encephalopathy (BASE), differs from classical BSE for the occurrence of a distinct type of the disease-associated prion protein (PrP), termed PrP^Sc^, and the presence of PrP amyloid plaques. Here, we show that the agents responsible for BSE and BASE possess different biological properties upon transmission to transgenic mice expressing bovine PrP and inbred lines of nontransgenic mice. Strikingly, serial passages of the BASE strain to nontransgenic mice induced a neuropathological and molecular disease phenotype indistinguishable from that of BSE-infected mice. The existence of more than one agent associated with prion disease in cattle and the ability of the BASE strain to convert into the BSE strain may have important implications with respect to the origin of BSE and spongiform encephalopathies in other species, including humans.

## Author Summary

Twenty years after the identification of bovine spongiform encephalopathy (BSE), the origin of the causal agent is still unknown. This issue is of fundamental importance, since knowledge of the origin of the BSE agent is essential for prevention of future outbreak of the disease, or variants thereof, in cattle and other mammals. In this study, we show that an atypical form of spongiform encephalopathy of cattle, termed BASE, is caused by a prion strain distinct from that of classical BSE. Noteworthy, this newly characterized prion strain has the ability to convert into the classical BSE strain upon serial transmission to inbred mouse lines. According to these results, BASE, which is regarded as a sporadic form of prion disease in cattle, may be the origin of BSE, following conversion of the causal agent in an intermediate host. These findings may have major implications with respect to the origin of BSE epidemic and spongiform encephalopathies in other species, including humans.

## Introduction

The transmissible spongiform encephalopathies (TSE), or prion diseases, are a group of neurodegenerative disorders that include Creutzfeldt-Jakob disease (CJD) in humans, scrapie in sheep, and spongiform encephalopathy in cattle (BSE) [1]. The central event in TSE pathogenesis is the conformational conversion of the cellular prion protein (PrP^C^) into insoluble, protease-resistant forms, termed PrP^Sc^, which accumulate in the brain [2]. According to the protein-only hypothesis [2], PrP^Sc^ isoforms are responsible for disease transmissibility by converting PrP^C^ into PrP^Sc^ [1–4].

There are several strains of the agents that cause TSE that can be distinguished by the disease characteristics they produce in experimentally infected animals, in particular the incubation periods and neuropathological profiles when passaged into panels of inbred mouse lines [5,6]. While multiple agent strains have been identified in natural scrapie [5–7] and CJD [8,9], early evidence indicated that BSE was caused by a single strain [10,11] having the ability to cross the species barriers quite efficiently and showing stable features even when transmitted to other species. Accidental transmission has occurred in cats, exotic ungulates, carnivores in zoological parks, and humans who developed a variant form of Creutzfeldt-Jakob disease (vCJD) [11–13]. These BSE-related disorders share a PrP^Sc^ type identical to that of BSE with regard to both the size of the protease-resistant core and the glycoform ratio, marked by predominance of the high molecular mass glycoform.

In the last few years, two atypical neuropathological and molecular phenotypes of BSE have been recognized in different European countries and Japan through active surveillance systems [14–17], and recent data indicate that they are linked to separate prion strains [18,19]. One of these phenotypes has been identified in Italy in two cattle aged 15 and 11 y and is distinguishable from BSE for striking differences in pattern of deposition and brain regional distribution of PrP^Sc^, with presence of prion protein (PrP) immunoreactive amyloid plaques and severe involvement of the olfactory system with relative sparing of the brainstem, as opposed to typical BSE [16]. The molecular signature of this “amyloidotic” form of bovine spongiform encephalopathy, named BASE, is a PrP^Sc^ type having a protease-resistant core of lower molecular mass than BSE-PrP^Sc^ with predominance of the monoglycosylated species [16]. To investigate the characteristics of the agent strains responsible for BSE and BASE, we carried out transmission studies using transgenic mice expressing bovine PrP and various lines of nontransgenic mice. We found that BSE and BASE are caused by prion strains having distinct biological properties and that the BASE strain is able to convert into the classical BSE strain upon serial transmission to inbred mouse lines.

## Results

### Transmissions of BSE and BASE to Transgenic Mice Expressing Bovine PrP

**Figure 1:**
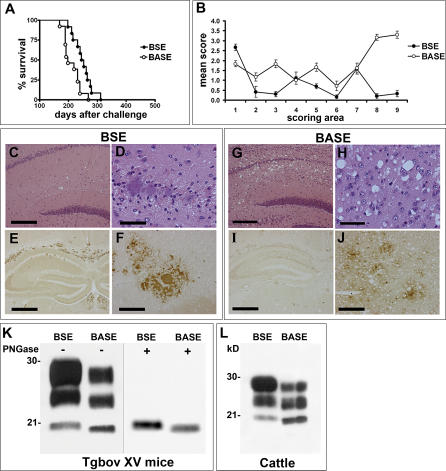
Primary Transmission of BSE and BASE to Tgbov XV Mice (A) Survival curves and (B) lesion profiles for mice infected with BSE and BASE. (B) Vacuolation was scored on a scale of 0–5 in the following brain areas: 1, dorsal medulla; 2, cerebellar cortex; 3, superior colliculus; 4, hypothalamus; 5, thalamus; 6, hippocampus; 7 septum; 8, retrosplenial and adjacent motor cortex; and 9, cingulate and adjacent motor cortex. Data are mean ± s.e.m. (C–J) Neuropathological changes in mice infected with BSE (C–F) and BASE (G–J). Micrographs were obtained from corresponding areas of the hippocampal region and cerebral cortex stained with haematoxylin-eosin (C, D, G, and H), or labeled with the anti-PrP antibody 6H4 (E, F, I, and J). The neuropathological profile of BSE-infected mice is marked by the presence of numerous amyloid plaques while spongiform changes are mild (C and D). Conversely, mice challenged with BASE show a severe vacuolation in the absence of amyloid deposits (G and H). PrP immunohistochemistry shows uni- and multicentric amyloid plaques associated with granular deposits in BSE-infected mice (E and F) and diffuse immunostaining of the neuropil with focal enhancement in mice challenged with BASE (I and J). Scale bar: 200 μm (C and G); 50 μm (D and H); 500 μm (E and I); 100 μm (F and J). (K and L) Western blot analysis of proteinase K-treated brain homogenates from (K) Tgbov XV mice challenged with BSE and BASE, prior (left panel) and after (right panel) deglycosylation with PNGase; and (L) cattle with BSE and BASE. The blots were probed with the anti-PrP antibody 6H4. The samples in (L) correspond to the actual inocula used for the transmission studies.

**Figure 2:**
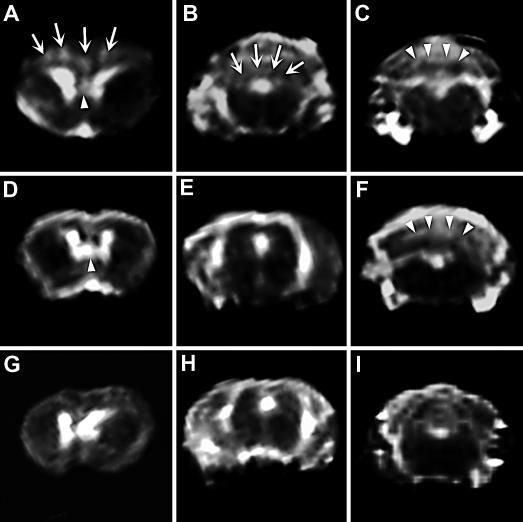
Brain MRI of Tgbov XV Mice Infected with BSE and BASE T2-weighted images of anterior-to-posterior coronal planes of (A–C) BASE-infected mouse; (D–F) BSE-infected mouse; (G–I) uninfected Tgbov XV mouse. Both BSE- and BASE-infected mice show high signal intensity in the septal region: arrowheads in (A) and (D); and cerebellum: arrowheads in (C) and (F) compared to control (G) and (I). In addition, mice challenged with BASE exhibit scattered hyperintense areas in frontal regions: arrows in (A); and midbrain: arrows in (B) that are absent in BSE-infected and uninfected mice.

Transgenic mice overexpressing bovine PrP on a murine PrP knockout background (Tgbov XV) [20] were inoculated both intracerebrally and intraperitoneally with BSE and BASE brain homogenates that were prepared from cattle having the same PrP genotype and comparable amount of PrP^Sc^ (Figure 1). Both BSE and BASE transmitted readily to these mice, but produced different clinical, neuropathological, and molecular disease phenotypes. The incubation period and survival time were significantly shorter (*p* = 0.006, log-rank test) in mice infected with BASE (means ± standard error of the mean [s.e.m.]: 178 ± 6 and 211 ± 8 d) compared to BSE (means ± s.e.m: 216 ± 10 and 244 ± 8 d) (Figure 1A). The initial clinical signs of disease consisted in lethargy in BASE-infected mice as opposed to hyperexcitability in BSE-infected mice. In vivo MRI of selected symptomatic animals revealed a different distribution of signal abnormalities on T2-weighted images. While both groups showed hyperintensity in the septal area and cerebellum, only BASE-infected mice had signal changes in frontal regions and midbrain (Figure 2). Neuropathological examination showed striking differences in “lesion profile” (i.e., the extent of vacuolar degeneration in standard brain regions) [21] with major involvement of somatosensory cortex and superior colliculus in BASE-infected mice as opposed to substantial sparing of these regions in BSE-infected mice (Figure 1B). Furthermore, in most scoring areas, the severity of vacuolar degeneration was remarkably higher in experimental BASE than in BSE (Figure 1B, 1C, 1G, and 1H). On the other hand, BSE-infected mice had amyloid plaques in the stratum oriens of the hippocampal formation, corpus callosum, and, to a lesser extent, in the septal region and brainstem (Figure 1D), whereas amyloid deposits were absent in BASE-infected animals as revealed by specific staining for amyloid. Immunohistochemistry showed that uni- and multicentric PrP plaques in BSE mice were associated with substantial amounts of granular PrP deposits in the same areas, while diffuse PrP immunoreactivity was weak (Figure 1E and 1F). At variance, BASE-infected mice showed primarily diffuse PrP deposition in the cerebral cortex, particularly in the somatosensory region, basal ganglia, thalamus, hypothalamus, brainstem, and cerebellum, accompanied by focal (granular, perivacuolar, and perineuronal) PrP deposits (Figure 1I and 1J). The phenotypic differences between experimental BASE and BSE were paralleled by differences in biochemical type of PrP^Sc^ similar to those found in cattle, both with respect to fragment sizes and glycoform ratio (Figure 1K and 1L). In particular, PrP^Sc^ from BASE-infected mice had a protease-resistant core of lower molecular mass than mouse-BSE PrP^Sc^ and showed a predominance of the monoglycosylated rather than the diglycosylated species (Figure 1K).

### Primary Transmissions of BSE and BASE to Inbred Mouse Strains

**Figure 3:**
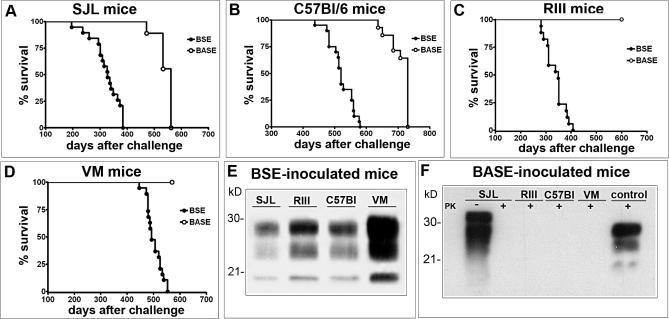
Survival Curves and PrP^Sc^ Types for Inbred Mice Following Primary Transmission of BSE and BASE (A–D) Survival curves are for (A) SJL mice, (B) C57Bl/6 mice, (C) RIII mice, and (D) VM mice. (E) Western blot analysis of proteinase K-treated brain homogenates from SJL, RIII, C57Bl/6, and VM mice challenged with BSE. (F) Western blot analysis of brain homogenates from SJL, RIII, C57Bl/6, and VM mice challenged with BASE and a BSE-infected SJL mouse used as positive control (last lane). The first two lanes correspond to the same SJL mouse prior to and after proteinase K digestion, while all other samples were digested with proteinase K. The blots were probed with the anti-PrP antibody 6H4.

**Figure 4:**
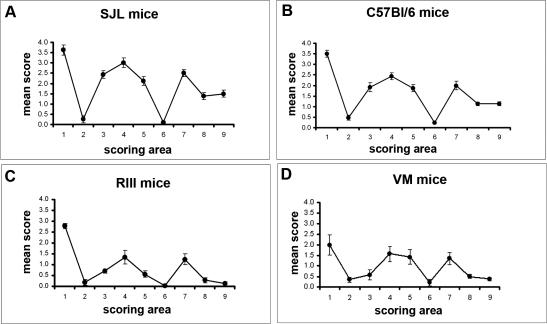
Lesion Profiles for Inbred Mice Following Primary Transmission of BSE (A–D) Lesion profiles are for (A) SJL mice, (B) C57Bl/6 mice, (C) RIII mice, and (D) VM mice. The lesion profile was determined as specified in Figure 1. Data are mean ± s.e.m.

The same BSE and BASE inocula used for Tgbov XV mice were injected intracerebrally and intraperitoneally into panels of four inbred mouse strains, including SJL, C57Bl/6, RIII, and VM mice. All mouse lines challenged with BSE developed a progressive neurological disease, according to previous reports [10,11,22]. The shortest incubation period was seen in SJL mice with a mean (± s.e.m.) of 273 ± 4 d, followed by RIII mice (313 ± 9 d), C57Bl/6 mice (437 ± 5 d), and VM mice (497 ± 6 d) (Figure 3A–3D). Neuropathologic examination of BSE-infected SJL, C57Bl/6, RIII, and VM mice sacrificed at the terminal stage of disease revealed a spongiform encephalopathy with preferential involvement of the medulla oblongata, hypothalamus, and septum. Although the lesion profile was similar in all mouse lines, the severity of vacuolar degeneration was greater in SJL and C57Bl/6 than in RIII and VM mice (Figure 4). Immunohistochemistry showed PrP^Sc^ immunoreactivity in the form of diffuse and focal (granular and plaque-like) deposits in the somatosensory cortex, striatum, thalamus, hypothalamus, septum, cerebellum, medulla oblongata, and spinal cord. The plaque-like PrP deposits did not possess the tinctorial properties of amyloid and were most abundant in VM mice. Immunoblot analysis of brain homogenates revealed the same PrP^Sc^ profile in all mouse lines, with predominance of the high molecular mass glycoform and a protease-resistant fragment similar to that of BSE (Figure 3E).

In contrast, SJL and C57Bl/6 mice injected with BASE did not develop clinical signs of neurological dysfunction and were culled near their predicted life span (SJL between 480 and 560 d and C57Bl/6 between 680 and 730 d after challenge) (Figure 3A and 3B). At the time of writing, transmission of BASE to RIII and VM mice has been in progress for 600 d, and no neurological signs of disease have yet been observed in any mice (Figure 3C and 3D). No PrP^Sc^ was detected by immunohistochemistry and Western blot analysis in the brain of the SJL and C57Bl/6 mice inoculated with BASE and in two RIII mice and two VM mice culled between 506 and 520 d after challenge (Figure 3F). The immunoblots were negative even when the analysis was carried out using 300–500 μl of 10% brain homogenate following phosphotungstic acid precipitation—i.e., a procedure that enables us to concentrate PrP^Sc^ [23]—with the exception of one RIII mouse that showed a PrP^Sc^ type identical to that of BSE-infected mice.

### Second-Passage Transmissions of BSE and BASE to Inbred Mouse Strains

**Figure 5:**
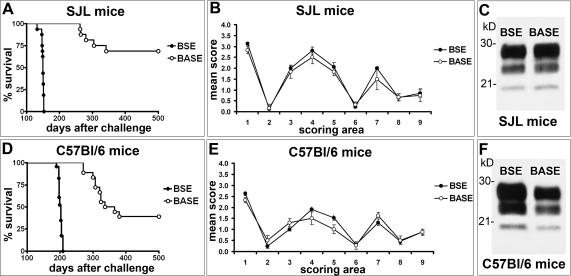
Survival Curves, Lesion Profiles, and PrP^Sc^ Types for Inbred Mice Following Second-Passage Transmission of BSE and BASE (A and D) Survival curves are for (A) SJL mice and (D) C57Bl/6 mice. (B and E) Lesion profiles are for (B) SJL mice and (E) C57Bl/6 mice. The lesion profile was determined as specified in Figure 1. Data are mean ± s.e.m. (C and F) Western blot analysis of proteinase K-treated brain homogenates from SJL mice (C) and C57Bl/6 mice (F) after challenge with BSE and BASE. The blots were probed with the anti-PrP antibody 6H4.

As a subsequent step in our study, we performed second-passage transmissions of brain homogenates from SJL and C57Bl/6 mice challenged with BSE and BASE. SJL mice inoculated with SJL-derived BSE agent developed disease with incubation period and survival time of 128 ± 2 and 149 ± 1 d (mean ± s.e.m.) (Figure 5A). A similar onset anticipation of disease on second passage compared to primary transmission was observed in C57Bl/6 mice challenged with C57Bl/6-derived BSE agent, with mean incubation period and survival time (± s.e.m.) of 180 ± 1 and 201 ± 1 d (Figure 5).

**Figure 6:**
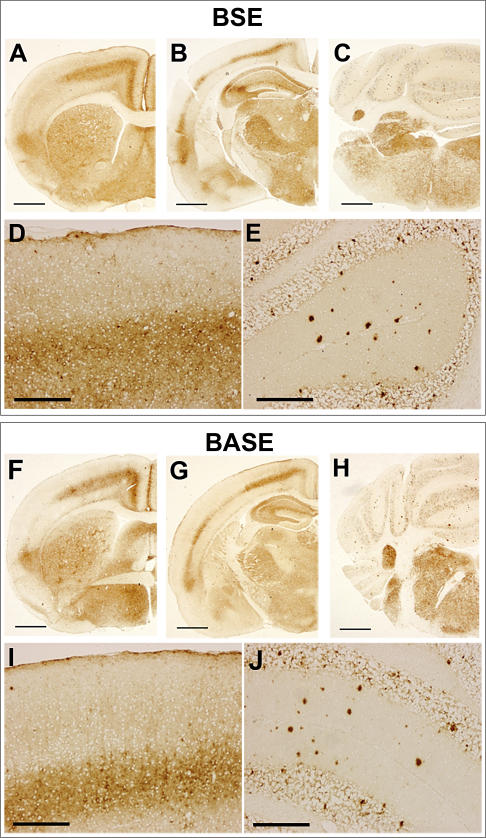
Immunohistochemical Detection of PrP in Brains of C57Bl/6 Mice Following Second-Passage Transmission of BSE and BASE (A–E) Mice infected with mouse-derived BSE or (F–J) mouse-derived BASE show the same brain regional distribution and patterns of deposition of PrP^Sc^. Micrographs were obtained from anterior-to-posterior coronal sections of the brain (A–C and F–H) and from corresponding areas of cerebral cortex (D and I) and cerebellum (E and J) probed with the anti-PrP antibody 6H4. Scale bar: 1 mm (A–C and F–H); 200 μm (D, E, I, and J).

At present, transmissions from BASE-inoculated SJL and C57Bl/6 mice, culled 560 and 730 d after primary challenge, respectively, have been in progress for 580 d. Noteworthy, 25% of SJL mice (4/16) and 60% of C57Bl/6 mice (11/18) have developed clinical signs of disease, with incubation periods in individual animals ranging from 237 to 323 d (mean ± s.e.m: 270 ± 16) and from 258 to 331 d (mean ± s.e.m: 299 ± 7), respectively (Figure 5A and 5D). All symptomatic BSE- and BASE-infected animals from the second passage were subjected to neuropathological examination and Western blot analysis at the terminal stage of disease. The lesion profile, as well as the pattern of deposition, brain regional distribution, and biochemical type of PrP^Sc^ in SJL and C57Bl/6 mice challenged with mouse-derived BSE, was similar to those observed in primary transmission. In particular, the vacuolar changes were most severe in the medulla oblongata, hypothalamus, and septum (Figure 5B and 5E). PrP immunoreactivity in the form of diffuse and focal deposits was mainly localized in the somatosensory cortex, striatum, thalamus, hypothalamus, septum, cerebellum, and medulla oblongata (Figure 6A–6E), and the PrP^Sc^ profile was marked by a protease-resistant fragment similar to that of cattle BSE with predominance of the high molecular mass glycoform (Figure 5C and 5F). Surprisingly, identical pathological changes, including lesion profile (Figure 5B and 5E), pattern of PrP immunoreactivity (Figure 6F–6J), and PrP^Sc^ type (Figure 5C and 5F) were found in clinically affected SJL and C57Bl/6 mice challenged with mouse-derived BASE.

## Discussion

Our study shows that transgenic mice expressing bovine PrP are highly susceptible to infection with both BSE and BASE. Experimental transmissions resulted in two neurological syndromes, each characterized by distinctive clinical features and brain regional distribution of MRI changes, arguing for propagation of two prion strains. The BSE and BASE isolates produced different incubation periods and showed differential characteristics in distribution of spongiform changes and patterns of PrP^Sc^ deposition, while retaining the PrP^Sc^ conformation of the respective inoculum. These findings are highly consistent with those obtained by another group following inoculation of the same Tgbov XV mouse line with different BSE and BASE isolates [19]. Also, in this instance, the BASE-infected mice did not show PrP amyloid plaques, at variance with cattle affected by natural BASE, confirming that the occurrence of a neuropathologic phenotype different from that of the donor is not an uncommon feature in transgenic mice [24]. Noteworthy, the BASE strain was more aggressive in Tgbov XV mice than the BSE strain, as deduced by the significantly shorter incubation time and the higher severity of lesion burden with appearance of the “hippocampal signature,” i.e., marked vacuolation of the superior region of Ammon's horn [25]. The complete homology of the PrP sequence between donor animals, i.e., BSE and BASE cattle [16] and the Tgbov XV recipient mice [20] speaks in favor of distinct PrP^Sc^ conformers being responsible for divergent phenotypic effects upon transmission. Overall, these results indicate that BSE and BASE are distinct diseases caused by different prion strains.

In contrast to intraspecies transmission, passage of TSE agents from one species to another is limited by the “species barrier” that may result in unsuccessful or inefficient transmission, with increased incubation periods, decreased fraction of animals succumbing to disease during their life span, and occurrence of subclinical infection [26–28]. Following primary inoculation of BASE into various inbred mouse strains, no clinical, neuropathological, or molecular signs of disease were observed in any animals, except one RIII mouse that showed trace amounts of PrP^Sc^ having biochemical properties identical to those of BSE-infected mice. These results cannot be accounted for by low infectivity titre, since the same BASE inoculum was used for primary transmission to Tgbov XV mice that showed a 100% attack rate. Instead, our findings indicate the presence of a substantial barrier to transmission of BASE to inbred mice with the occurrence of subclinical infection, at variance with BSE that was easily transmitted on primary passage, in agreement with previous reports [10,11]*.* Overall, these data support the conclusions drawn from transmissions to Tgbov XV mice that the BASE strain is distinct from the BSE strain.

The species barrier effects are usually reduced and stabilized on further passages, whereas other strain-encoded biological properties, including disease-associated PrP^Sc^ type and brain lesion profile, remain unchanged regardless of incubation times. However, in some instances, the occurrence of temporary (“only at first passage”) and permanent (“selection of mutant”) modifications of prion strains have been demonstrated following interspecies transmission of mouse scrapie strains [29]*.* Recently, transmission of human vCJD prion strain to mice with chimeric human/mouse PrP has resulted in the isolation of two stable prion strains with distinct incubation times and different sizes of the protease-resistant core of PrP^Sc^ [30]*.* In line with these results, experimental transmission of BSE isolates to transgenic mice expressing human PrP with methionine at codon 129 produced two distinct PrP^Sc^ types and neuropathological phenotypes, or propagated two distinct PrP^Sc^ glycotypes in inbred mouse strains [22,31]*.* These findings have suggested the presence of two prion strains in individual cases of vCJD and cattle BSE, or alternatively, the generation of a new prion strain. Whatever the explanation, experimental proof exists that interspecies transmission of the BSE/vCJD strain may result in the birth or isolation of a new prion.

A striking finding of our study was the observation of converging features, including PrP^Sc^ type and neuropathological profile (but not incubation period) of BASE-subpassaged inbred mice toward BSE-inoculated mice. In this respect, the BASE strain differs from the prion strain associated with distinct atypical cases of BSE recognized in France, whose molecular features are maintained upon transmission to C57Bl/6 mice [18]. Conversion of a prion strain into another has been previously observed upon transmission of the scrapie agent 87A to C57Bl mice. The newly generated prion strain, termed 7D, has biological characteristics, including incubation period and brain lesion profile, similar to those of the Me7 prion strain [32]. The present demonstration that transmission of BASE to inbred mice can propagate a prion strain, which generates a neuropathological profile and a PrP^Sc^-type similar to BSE-inoculated animals, appears strictly complementary to previous observations documenting a prion dichotic switch in BSE/vCJD-infected mice [22,30]. We consider our data inconsistent with a cross-contamination of BASE homogenates with BSE for a number of reasons, including (i) the strict biosafety procedures used for sample collection, preparation of the inocula, inoculation scheme, and care of mice; (ii) the absence of BSE-type PrP^Sc^ in the BASE inocula used for primary- and secondary-passage transmissions as deduced by Western blot analysis; (iii) the divergence between absence of BSE-type PrP^Sc^ in the inocula and the short incubation period observed on second passage of BASE to SJL and C57Bl/6 mice as compared to primary transmission of BSE to the same mice; and (iv) the absence of disease in control mice inoculated with normal brain homogenates prepared with the same protocols. Instead, our data are consistent with host-induced modification of the properties of the BASE strain upon transmission to inbred mice. The finding of trace amounts of PrP^Sc^ in one RIII mouse following primary challenge with BASE suggests that these modifications occurred on first passage.

The origin of BSE—an issue of fundamental importance for the control of disease—is still unknown. Demographic and incidence data suggest that BASE could represent a sporadic form of cattle prion disease [16], pre-existing the appearance of BSE epidemics. This view is supported by the observation that the natural cases of BASE identified so far affected old cattle, despite the fact that the BASE prion is more aggressive than the BSE prion for transgenic mice expressing bovine PrP and cattle experimentally infected by intracerebral route (G. Lombardi, unpublished data). Lack of recognition of BASE in the past can be tentatively explained by the paucisymptomatic syndrome observed in original cases [16] and in experimentally BASE-infected cattle that show only reduced alertness and weight loss (G. Lombardi, unpublished data), consistent with the features of Tgbov XV-infected mice. Therefore, the hypothetical scenario that can be envisaged is that the BSE agent could have originated from the BASE strain, following conversion in peripheral tissues or in an intermediate host.

Although BASE showed a significant barrier to primary transmission to inbred mouse lines resulting in preclinical infection, it remains of considerable concern whether the causal agent is potentially pathogenic for humans. In this regard, the neuropathological and molecular features of BASE—in particular, the biochemical type and deposition patterns of PrP^Sc^—have striking similarities with those of a distinct subgroup of patients with sporadic CJD [16]. This finding requires a cautious interpretation. Nonetheless, the possibility that the origin of CJD in some patients with a sporadic disease phenotype may be related to BASE exposure has to be considered and verified through transmission studies.

## Materials and Methods

### Transmission studies.

Four inbred mouse strains (SJL, RIII, C57Bl/6, and VM) and transgenic mice overexpressing bovine PrP with six octapeptide repeats on a murine PrP knockout background (Tgbov XV) [20] were used for the study. Groups of 15–20 mice of each strain were anaesthetized with sevofluorane and injected by a combination of intracerebral (20 μl) and intraperitoneal (100 μl) routes with 10% homogenates prepared from the thalamus of two cattle: one BSE-affected (code 128204) and one BASE-affected (code 1088). [16]. The two cattle had the same PrP genotype with six octapeptide repeats, and the BSE and BASE inoculum contained comparable amount of PrP^Sc^, as deduced by Western blot analysis with the antibody 6H4. Groups of uninjected mice and mock-inoculated mice of each strain were included as controls. Mice were unequivocally identified with ear-tags and housed in groups of four animals in individually ventilated cages, except the SJL mice that were single-housed due to aggressive behavior. Behavioral monitoring was carried out weekly and included spontaneous locomotor activity in the open field, reactivity to external stimuli, nest construction test, and inverted screen test [33–35]. The incubation time was calculated as the period between the day of inoculation and the appearance of clinical signs of disease, confirmed by a subsequent assessment at a 1-wk interval. Clinically affected mice were sacrificed at the terminal stage of disease while all other mice were monitored for the entire predicted life span and then culled and subjected to autopsy. For the second passage to SJL and C57Bl/6 mice, the inocula were prepared from pools of brains of terminally BSE-sick SJL and C57Bl/6 mice, brains of SJL and C57Bl/6 mice challenged with BASE and sacrificed at the end of their life span, and brains of uninoculated SJL and C57Bl/6 mice used as negative controls. In both primary- and second-passage transmissions, particular attention was taken to avoid cross-contamination. In particular, the collection of samples and preparation of the inocula were carried out using sterile instrumentation and disposable equipment for each animal and each inoculum. Furthermore, inoculation of the experimental groups with the BASE strain was always performed before that with the BSE strain, and the animals were housed in separate IVC racks. The procedures involving animals and their care were conducted in conformity with national and international laws and policies (EEC Council Directive 86/609, OJ L358, 1, 12 December 1987; Italian Legislative Decree 116/92, Gazzetta Ufficiale della Repubblica Italiana 10, 18 February 1992; and *Guide for the Care and Use of Laboratory Animals*) and the study was approved by the authors' Institutional Review Boards.

### Neuropathology.

At autopsy, the left lateral two-thirds of each mouse brain was fixed in Carnoy solution at 4 °C for 24 h [36], while the right third of each brain was frozen at −80 °C for Western blot analysis and transmission studies. The Carnoy-fixed samples were dissected at four standard coronal levels [21], dehydrated, and embedded in paraplast. 5-μm thick serial sections from paraplast-embedded blocks were stained with hematoxylin-eosin (HE), Nissl and thioflavine S for amyloid, or probed with anti-PrP antibodies. The lesion profiles were determined on HE-stained sections, by scoring the vacuolar changes in nine standard grey matter areas as described [21]. PrP immunoistochemistry was carried out using the monoclonal antibody 6H4 (Prionics, http://www.prionics.com) [37] and the polyclonal antibody PrP95–108 [38]. Before immunostaining, sections were pretreated with proteinase K followed by guanidine thiocyanate as reported previously [36]. Immunoreactions were revealed with the Animal Research Kit Peroxidase (Dako, http://www.dako.com) for 6H4 and the polyclonal EnVision system (Dako) for PrP95–108, using 3–3′-diamonobenzidine as chromogen.

### Protein analysis.

Frozen samples were homogenized in nine volumes of cold lysis buffer (100 mM sodium chloride, 10 mM EDTA, 0.5% Nonidet P-40, 0.5% sodium deoxycholate in 10 mM Tris HCl [pH 7.4]). Aliquots equivalent to 250-μg protein were digested with 50 μg/ml proteinase K (PK; Boehringer Mannheim, http://www.boehringer.com) at 37 °C for 1 h. After PK digestion, some samples were deglycosylated with recombinant peptide N-glycosidase F (New England Biolabs, http://www.neb.com) at 37 °C for 12 h following manufacturer's instruction. The samples were fractionated by 13% Tris-tricine-sodium dodecyl sulphate-polyacrylamide gel electrophoresis under reducing conditions, electrophoretically transferred to polyvinylidene difluoride membranes (Immobilon; Millipore, http://www.millipore.com), and probed with the monoclonal antibody 6H4 (1:5,000) as described [16]. Blots were developed using enhanced chemoluminescence system (Amersham, http://www.gehealthcare.com) and visualized on autoradiography films. To enhance PrP^Sc^ detection, samples that were negative on standard immunoblot were subjected to phosphotungstic acid (PTA) precipitation as described [23] and analyzed by Western blot.

### Magnetic resonance imaging.

Magnetic resonance imaging was performed in clinically symptomatic Tgbov XV mice challenged with BSE and BASE and in noninfected control mice (*n* = 3 animals/group) on a Siemens Magnetom Avanto 1.5 Tesla scanner. The body coil was used for transmission, and a quadrature surface coil was used as receiver. Animals were anaesthetized with tribromoethanol (Avertin) and placed in a closed holder avoiding any biological contact with the environment and providing filtered ventilation; temperature and breathing were monitored throughout the procedure. Sixteen coronal slices were acquired in each mouse with T2 turbo spin-echo sequences; the following parameters were employed: thickness 1.5 mm without gap, TR = 4,000 ms, TE = 108 ms, 44.8 mm square FoV, 128 × 128 matrix, and 14 averages.

### Statistical analysis.

Statistical analysis was performed using the GraphPad-Prism software. Kaplan-Meier survival curves were plotted, and differences in survival between groups of mice inoculated with BSE and BASE were compared using the log-rank test.

## Abbreviations

BASE: bovine amyloidotic spongiform encephalopathy
BSE: bovine spongiform encephalopathy
CJD: Creutzfeldt-Jakob disease
PrP: prion protein
PrP^Sc^: disease-associated form of PrP
s.e.m.: standard error of the mean
Tgbov XV mice: transgenic mice expressing bovine PrP
TSE: transmissible spongiform encephalopathy
vCJD: variant CJD
